# Effect of Eudragit S100 nanoparticles and alginate chitosan encapsulation on the viability of *Lactobacillus acidophilus* and *Lactobacillus rhamnosus*

**DOI:** 10.1186/s13568-017-0442-x

**Published:** 2017-07-06

**Authors:** Fereshteh Ansari, Hadi Pourjafar, Vahid Jodat, Javad Sahebi, Amir Ataei

**Affiliations:** 1 Research Center for Evidence Based Medicine, Tabriz University of Medical Sciences, Tabriz, Iran; 2 Department of Public Health, Maragheh University of Medical Sciences, Maragheh, Iran; 3 Tabriz Branch, Islamic Azad University, Tabriz, Iran

**Keywords:** Encapsulation, *Lactobacillus acidophilus*, *Lactobacillus rhamnosus*, Viability, Chitosan, Eudragit S100 nanoparticles

## Abstract

In this study, we examined a novel method of microencapsulation with calcium alginate-chitosan and Eudragit S100 nanoparticles for the improving viability of probiotic bacteria, *Lactobacillus acidophilus* and *Lactobacillus rhamnosus*. Extrusion technique was carried out in microencapsulation process. The viability of two probiotics in single coated beads (with only chitosan), double coated beads (with chitosan and Eudragit nanoparticles), and as free cells (unencapsulated) were conducted in simulated gastric juice (pH 1.55, without pepsin) followed by incubation in simulated intestinal juice (pH 7.5, with 1% bile salt). In case of single coated beads, presumably, lack of sufficient strength of chitosan under simulated gastric condition was the main reason of 4-log and 5-log reduction of the counts of the *L. acidophilus* and *L. rhamnosus* respectively. The results showed that with the second coat forming (Eudragit nanoparticles) over the first coat (chitosan), the strength of the beads and then viability rate of the bacteria were increased in comparison with the single coated beads.

**Electronic supplementary material:**

The online version of this article (doi:10.1186/s13568-017-0442-x) contains supplementary material, which is available to authorized users.

## Introduction

The low viability of probiotics in difficult conditions, especially throughout the time of processing to consumption of food products and in the gastrointestinal (GI) conditions, has persuaded the researchers to find methods for improving the criteria. Microencapsulation has a significant effect in this case. Microencapsulation technologies have been extended and applied effectively to protect the probiotic bacteria from damages caused via the exterior milieu in the situations such as processing (high temperature), storage (in the food products on the shelf and in a home like foodstuff matrix), packaging (temperature, oxygen, humidity) and degradation in the GI region (the low pH in the stomach and bile salt in the small intestine) (Anal and Singh 2007; Moroeanu et al. 2015; Zuidam and Nedovic 2010).

One of the most basic materials that is used for probiotic microencapsulation is alginate. The main advantages of this material that make it more preferable over other materials for microencapsulation are its non-toxic to bacteria and body cells as an allowed additive, ease of use and cost-effectiveness. Although, this substance has some disadvantages for encapsulation purpose, such as sensibility, decomposition in acidic condition, decomposition in the presence of monovalent ions due to completion with calcium ions, quick moisture release, and other fluids form alginate. The disadvantages can be removed by creating a resistant coat on alginate or adding other chemicals to it, for example chitosan (Krasaekoopt et al. 2004, 2006; Rodklongtan et al. 2014).

Chitosan is a linear positive-charge (cationic) polysaccharide which is obtained from chitin and is used as a cover to strengthen microcoverage over another negative-charge microcovers. This substance, like alginate, is cost effective and harmless and creates a jell network (Li et al. 2015; Zuidam and Nedovic 2010). However, this material has some disadvantages, especially the lack of suitable strength in the acidic conditions, and therefor this substance detach during gastric passage and release it’s contain in the stomach (Badhana et al. 2013; Boeris et al. 2009; Quan et al. 2008).

Eudragit (Eu) S100 is an anionic copolymer derived from metacrylic acid and methyl metacrylate (1:2). It is non-soluble in acids and water, while it is soluble in a pH 7 solvent or higher alkaline circumstance (Higashi et al. 2015; Sharma et al. 2016). Eu polymers are nontoxic and food-grade polymers (Gibson et al. 2006; José 2006; Thakral et al. 2013). This material can help medicines to protect GI conditions and reach the colon, so it can be used as a secondary coverage for strengthening the microencapsulation as well as assuring targeting the release of probiotics in the colon, which is their major and functional place. Eu coating of chitosan capsules strengthens the beads and prevents the release of bacteria in the stomach. This improvement of bead’s stability may increase the count and viability of bacteria in the beads during GI lumen (Badhana et al. 2013; Boeris et al. 2009; Quan et al. 2008; Yoo et al. 2011). One important benefit of nanoparticles instead of Eu powder is the establishment of a thin nanosize layer around the beads. This very thin layer may improve the resistance of beads without enlarging the size of them (Pandey et al. 2016; Patel et al. 2015; Pourjafar et al. 2016; Yoo et al. 2011).

The goal of this study was to evaluate the effect of Eudragit S100 nanoparticles on the strengthen of calcium alginate and chitosan microcapsules to improve the viability of *Lactobacillus acidophilus* and *Lactobacillus rhamnosus* in the simulated gastrointestinal conditions.

## Materials and methods

### Preparation, activation and collection of probiotic bacteria

Probiotic bacteria cultures of *L. acidophilus* (PTCC 4356) and *L. rhamnosus* (PTCC 1469) were obtained from Iranian Research Organization for Science and Technology (IROST) and inoculated in MRS-broth (QUELAB, Canada) and incubated at 37 °C for 24 h in aerobic conditions. The probiotic bacteria growth in late-log phase was gathered by means of centrifugation (Eppendorf, Centrifuge 5810 R, Germany) at 3000*g* for 10 min., and afterward it was washed two times in sterilized distilled water prior to applying to the microencapsulation procedure. In free bacteria samples, after centrifugation, 1 ml distilled water added to the tubes (Ghorbani-Choboghlo et al. 2015; Pourjafar et al. 2016).

### Preparation of Eudragit S100 nanoparticles

Eu S100 powder was obtained from Evonik Pharma Polymers (Evonik, D-64275, Darmstadt, Germany). To prepare the Eu S100 nanoparticles, SAS (Supercritical Antisolvent Technique) progression was applied and the option of acetone (Scharlau Chemie S.A, Spain) was provided as a solvent for Eu S100 polymer. 4 mg ml^−1^ of Eu solution was infused into distilled water small quantity as a supercritical fluid that had been held beneath homogenizing force (Wisetise, DAIHAN Scientific Co., Ltd, Korea) at 9000*g* and at 35 °C for 10 min. Distilled water included 15 mg l^−1^ Tween 80 (Merk, Hohenbrunn, Germany) as a surfactant. Note that, the acetone solvent was left out through evaporation and particle size and Polydispersity Index (PDI) of Eu S100 was assessed via Laser Particle Size Analyzer device (Brookhaven Instruments Corporation, USA) (Hu et al. 2012; Yoo et al. 2011).

### Preparation of chitosan solution

A 0.4 g low-molecular-weight chitosan (Sigma, USA) was blended with 90 ml distilled water and acidified by means of 0.4 ml of glacial acetic acid (Merk, Darmstadt, Germany). Then, the pH was regulated in 5.6–5.8 by adding 1 mol (M) l^−1^ NaOH. The resulted solution of chitosan was filtered throughout Whatman #4 paper filter and the volume was adjusted to 100 ml before sterilizing at 121 °C for 15 min. Finally, this solution was refrigerated (5 °C) overnight (Abouhussein et al. 2016; Kanmani et al. 2011; Lee et al. 2004; Rodklongtan et al. 2014).

### Microencapsulation of probiotic bacteria and, single and double coating of beads

In this research, extrusion technique was carried out in the encapsulation process illustrated formerly via Krasaekoopt et al. (2004) and Pourjafar et al. (2012). A 4% sodium alginate (Sigma, USA) was blended with distilled water and then sterilized at 121 °C for 15 min, and refrigerated overnight. Following day, 10 ml of per bacterial suspension (2 × 10^10^ colony forming units (CFU/ml) was added in the sodium alginate solution. In the final step, the mixture of the cell suspension and sodium alginate were injected into sterile 0.1 mol l^−1^ CaCl_2_ (Merk, Darmstadt, Germany) solution using sterile insulin syringes (0.2 mm) as possible as we could pressure the syringe to force out the solution extremely fast. The droplets turned into gel spheres straight away (the distance between the CaCl_2_ solution and the needle was 20 cm), and after 60 min, all the beads were gathered and washed through distilled water (Mandal et al. 2006; Mirzaei et al. 2012; Pourjafar et al. 2012).

For the first coating, the beads were submerged in 100 ml of chitosan solution (0.4 g 100 ml^−1^) with gentle shaking at 1 g for 40 min on a magnetic stirrer (IKA Labortechnik, Model 79219 Staufen, KG, Germany). Following that, the chitosan single coated beads were gathered and washed through distilled water (Kanmani et al. 2011; Krasaekoopt et al. 2004; Liserre et al. 2007).

**Fig. 1:**
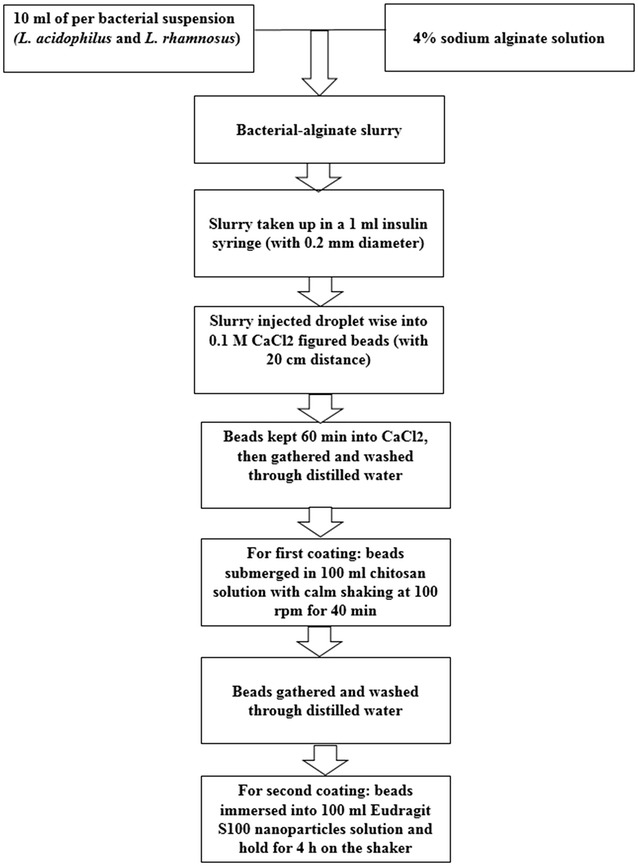
Schematic representation of the encapsulation of probiotic bacteria in calcium alginate chitosan and Eu S100 nanoparticles

In the final stage, for the second coating, the beads were immersed in 100 ml Eu S100 nanoparticles solution (4 mg 100 ml^−1^) and held for 4 h on the shaker (Badhana et al. 2013; Hu et al. 2012; Yoo et al. 2011). The double-coated beads were gathered and washed with distilled water and employed on the same day (see Fig. 1).

### Beads characterization

The diameters of 50 haphazardly picked beads (50 uncoated, 50 single coated and 50 double coated beads) were determined by means of an eyepiece micrometer on an optical microscope (Nikon-Model Alphaphot-2 YS2-T. Japan) at a magnification of 10× (magnification factor for each unit of gradient lens was 10.89 at magnification of 10×). The exterior morphology and the interior appearance of beads were examined using optical microscope at the magnifications of 40× and 100×. For examination of interior and exterior appearance, first we produced some beads larger than our normal beads we had produced (at about 1 mm). Then we prepared a cross section of beads by means of a microtome blade (for this purpose, we put beads on a plate and then divided them by microtome blade and by hand, then we applied gram staining on the cut surface) (Ghorbani-Choboghlo et al. 2015; Mirzaei et al. 2011, 2012). Moreover, Scanning Electron Microscope (SEM) method was used to differentiate between surfaces of the beads with or without a nanoparticles coating.

### Enumeration of free and microencapsulated probiotic bacteria

Free bacterial counts were determined by adding 1 ml solution containing bacteria in 9 ml phosphate buffer (0.1 mol l^−1^, pH 7.0) and 1 ml aliquot dilutions were dispensed onto the plates of the MRS-Salicin-agar (MRS agar; QUELAB, Canada and Salicin; Sigma, USA) for *L. acidophilus* and MRS-Glucose-vancomycin-agar (MRS agar; QUELAB, Canada, Glucose; Merk, Germany and Vancomycin; Sigma, USA) for *L. rhamnosus*. In MRS-Salicin-agar, Salicin (10 ml solution at 10% w/v) was added in 90 ml of sterilized MRS agar (Mirzaei et al. 2012; Sultana et al. 2000) and in MRS-Glucose-vancomycin-agar, glucose (10 ml solution at 10% w/v) and vancomycin (50 µg ml^−1^) were added in 90 ml of sterilized MRS agar (Saxelin et al. 2010).

All enumerated plates of *L. acidophilus* and *L. rhamnosus* were incubated at 37 °C for 48 h under aerobic condition. The averages were conveyed as colony forming units per ml of the sample (CFU ml^−1^). To enumerate the encapsulated probiotic bacteria, at first the captured bacteria were released from the beads. For this purpose, 1 g of the double coated beads were suspended in 9 ml of phosphate buffer (0.1 mol l^−1^, pH 7.0) after shaking for 60 min on a bag mixer (netech–laboratory, Bag Tech^®^) at room temperature (Pourjafar et al. 2016; Sultana et al. 2000).

### Survival of microencapsulated probiotic bacteria following sequential incubation in simulated gastric and intestinal juice (see Fig. 2)

This investigation was anchored in the technique expressed via Sultana et al. (2000), and especially Krasaekoopt et al. (2004) and Mirzaei et al. (2011). Single coated beads (1 g), double coated beads (1 g) and free bacteria suspension (1 ml) were separately placed in a tube including 9 ml of sterilized simulated gastric juice (0.08 mol l^−1^ HCl, including 0.2 g 100 ml^−1^ NaCl, pH 1.55) and incubated for 30, 60, 90, and 120 min at 37 °C. Subsequent to incubation, aliquots of 1 g of single coated beads or 1 g of double coated beads or 1 ml of free bacteria suspensions harvested from simulated gastric juice were added to 9 ml of sterilized simulated intestinal juice (0.05 mol l^−1^ KH_2_PO_4_, pH 7.5, with 1 g 100 ml^−1^ bile salt). Following that, these tubes were incubated for 150 min at 37 °C. After incubation time, beads were dissolved in phosphate buffer solution and cell count was assessed by “pour plate count method”. Also, in free bacteria sample, 1 ml of free bacteria suspension harvested from simulated intestinal juice was used for cell count by “pour plate count method” (see “Enumeration of free and microencapsulated probiotic bacteria”).

**Fig. 2:**
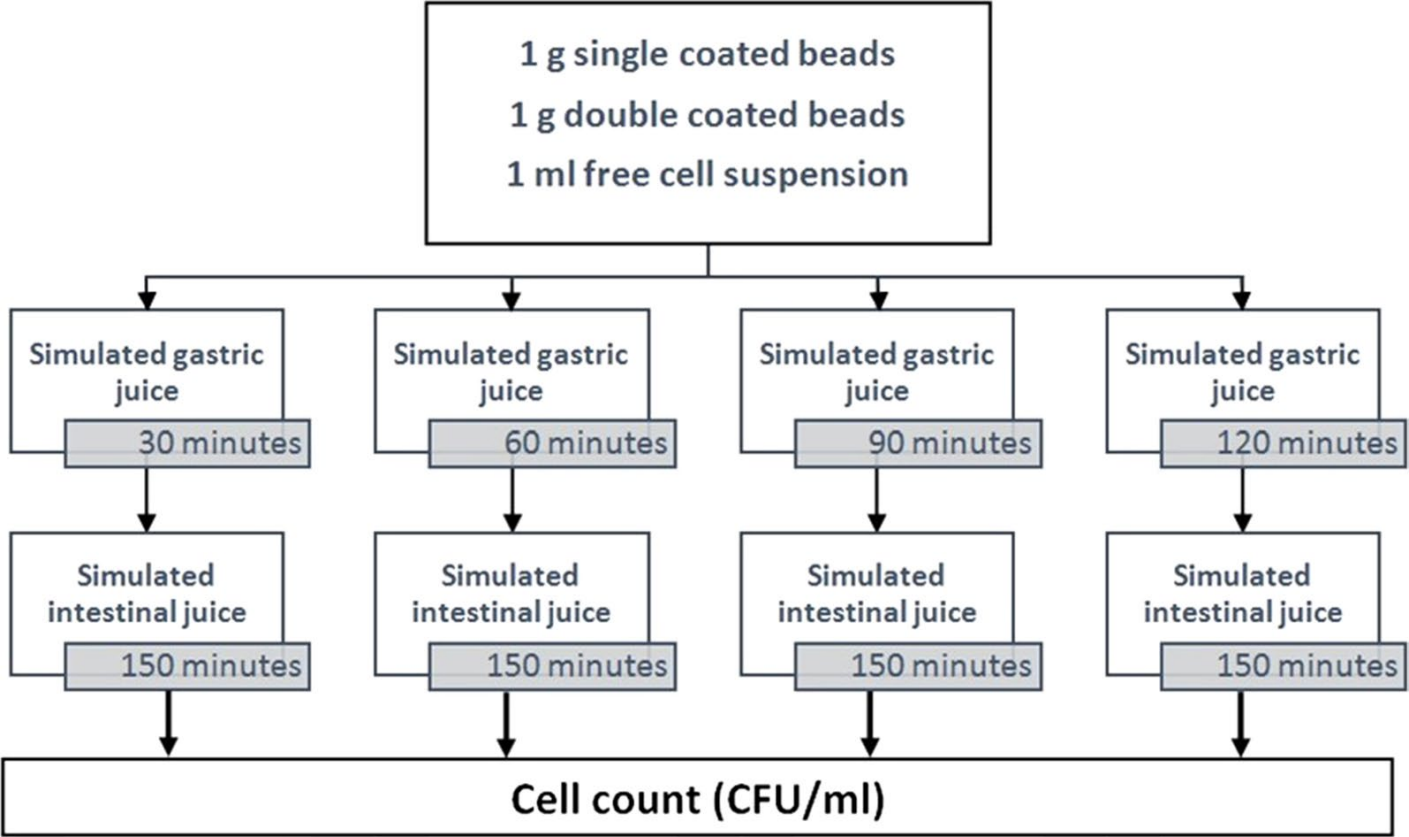
The diagram of the experimental process of survival of microencapsulated probiotic bacteria following sequential incubation in simulated gastric and intestinal juice

### Statistical analyses

The entire statistical analyses were carried out by means of SPSS 22 (IBM) software. The conducted tests were duplicate (n = 2). In the enumeration of the bacteria in each replication, plates with the colonies were counted and their internal concentration mean was used to prevent any internal error. Finally, the mean of two replications was calculated to remove external error. The number of the bacteria was reported in terms of the number of the colonies per 1 ml. After confirmation of normality of data by Kolmogrov-Smirnov test further analysis were carried out using Repeated Measures ANOVA test and P < 0.05 regarded to be significant. The graph (Fig. 5) has been constructed using GraphPad Prism version 6 software.

## Results

### Particle size of Eudragit S100 and morphology of beads

After preparation of the Eu S100 nanoparticles via SAS processing, the particle size and PDI of Eu S100 were achieved by means of Laser Particle Size Analyzer device. According to these analyses, particle size and PDI of Eu S100 particles were 100 nm and 0.410 respectively.

**Fig. 3:**
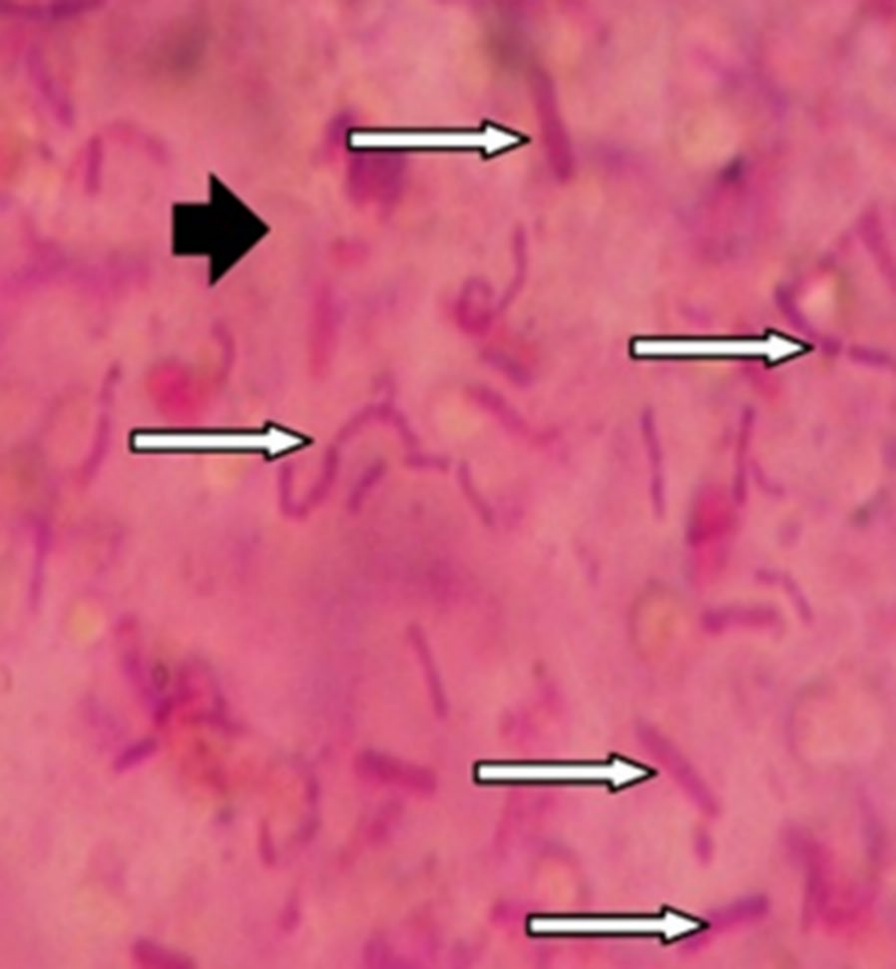
Vertical cross-section and internal appearance of bead at ×100 magnification after gram staining [see the positive gram bacteria (*rightwards black arrow*) are distributed randomly in the alginate matrix (*rightwards white arrow*)]

**Fig. 4:**
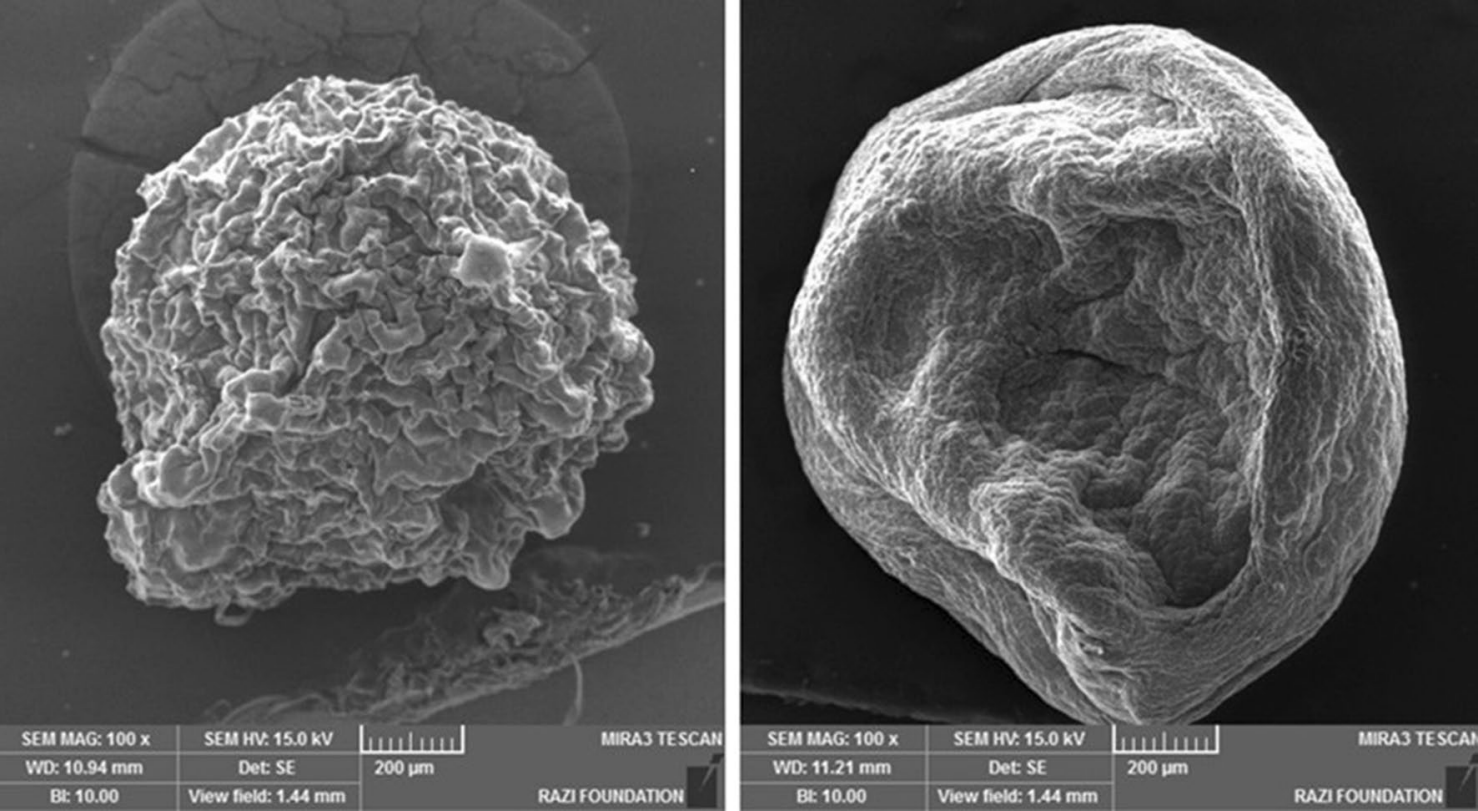
Scanning electron microscope photomicrographs of beads. Beads encapsulated form only with chitosan coating (*left*) and beads encapsulated form with chitosan and Eu S100 nanoparticles coating (*right*)

The mean ± standard deviation of diameters of the 50 haphazardly picked beads was 123.66 ± 41.73 µm. The interior appearance of the beads is shown in Fig. 3. The picture of the beads under an optical microscope (at 10× magnification) illustrated that the beads were sphere-shaped and the cross-section and inner appearance of beads (here we produced bigger beads at about 1 mm for showing clear figures) at 40× and 100× magnification illustrated that the bacterial cells were placed randomly in the alginate matrix. SEM was used for differentiating between surfaces of beads (at about 1 mm) with and without Eu nanoparticles coating (Fig. 4).

### Survival of probiotic bacteria following sequential incubation in simulated gastric and intestinal juice

**Fig. 5:**
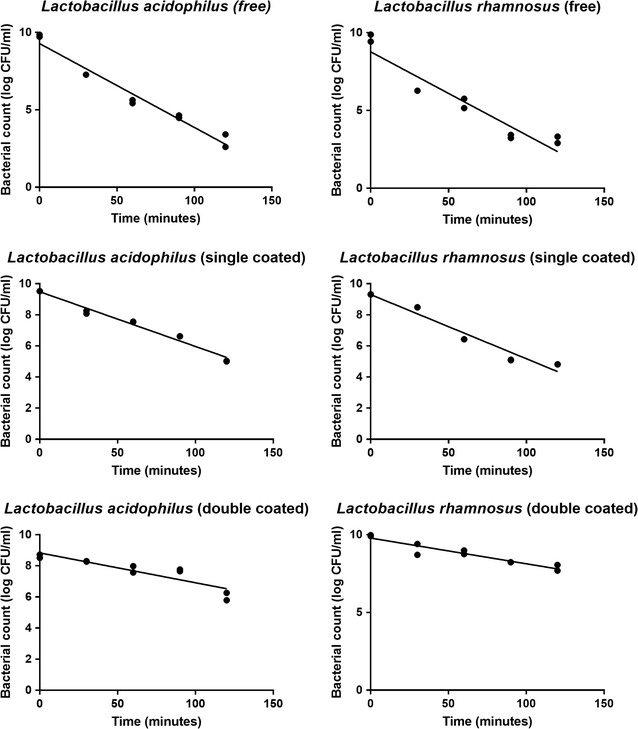
Cell survivability (log CFU ml^−1^) of three forms of *Lactobacillus acidophilus* and *Lactobacillus rhamnosus* (free, mono layer and double layer) after treatment in simulated gastric juice during 120 min (with 30 min interval) and then treatment in simulated intestinal juice with bile salt for 150 min (*Circles* represent bacterial count in each sample and the *line* is demonstrating the linear estimation of bacterial count)

To find out the effect of the acidic pH of the stomach and the effect of the intestinal juice on the viability rate of the microencapsulated probiotic bacteria, an in vitro schema was employed. For this purpose, the cultures of bacteria were deposited into the simulated gastric juice for 2 h, then into intestinal juice for 2.5 h. The outcomes are given in Table 1 and Fig. 5.

**Table 1:** Cell survivability (CFU/ml) of three forms of *Lactobacillus acidophilus* and *Lactobacillus rhamnosus* (free, mono layer and double layer) after treatment in simulated gastric juice during 120 min (with 30 min interval) and then treatment in simulated intestinal juice with bile salt for 150 min (mean of log count ± SD)

Bacteria	Form	0 min	30 min	60 min	90 min	120 min
*Lactobacillus acidophilus*	Fa	9.78 ± 0.09	7.27 ± 0.01	5.53 ± 0.14	4.56 ± 0.12	3.00 ± 0.57
	M1b	9.52 ± 0.00	8.17 ± 0.01	7.543 ± 0.02	6.60 ± 0.00	5.02 ± 0.03
	M2b	8.61 ± 0.15	8.28 ± 0.02	7.77 ± 0.29	7.70 ± 0.07	6.02 ± 0.33
*Lactobacillus rhamnosus*	Fa	9.65 ± 0.31	6.27 ± 0.01	5.45 ± 0.43	3.33 ± 0.14	3.11 ± 0.30
	M1b	9.31 ± 0.15	8.48 ± 0.01	6.43 ± 0.00	5.10 ± 0.02	4.82 ± 0.00
	M2c	9.93 ± 0.04	9.05 ± 0.49	8.87 ± 0.17	8.23 ± 0.00	7.87 ± 0.26

*F* free form, *M1* encapsulated form only with chitosan coating (single coating), *M2* encapsulated form with chitosan and Eudragit nanoparticles coating (double coating)

*a*, *b*, *c* The different lower case letters indicate statistically significant difference between declines in bacterial count between different bacterial form (statistical analysis was performed separately for each bacterial species)

The numbers of the free *L. acidophilus* at the time zero and after 120 min were 6.2 × 10^9^ and 1.5 × 10^3^ respectively. In addition, the number of this bacterium was reduced at about 6.7-log. In case of the free *L. rhamnosus*, the numbers of this bacterium at the time zero and after 120 min were 5.1 × 10^9^ and <1.0 × 10^2^ respectively. The reduction rate of this free bacterium’s count was 6.5-log (Additional file 1: Table S1).

In case of the single coated beads (coated only with chitosan) in 120 min, the number of the *L. acidophilus* reduced from 3.3 × 10^9^ to 1.1 × 10^5^ (the reduction rate was 4.5-log) and the number of the *L. rhamnosus* reduced from 2.1 × 10^9^ to 6.6 × 10^4^ (the reduction rate was 4.5-log) (Additional file 1: Table S1).

In double coated beads (with chitosan and Eu S100 nanoparticles), throughout 120 min period, the number of the *L. acidophilus* was reduced from 4.2 × 10^8^ to 1.8 × 10^6^ (the reduction rate was 2.6-log) and also, the number of the *L. rhamnosus* was reduced from 8.7 × 10^9^ to 8.1 × 10^7^ (the reduction rate was 2.1-log) (Additional file 1: Table S1).

## Discussion

In morphology of beads, comparable figures of the beads were also shown via Sultana et al. (2000), Krasaekoopt et al. (2004), Pourjafar et al. (2012), and Mirzaei et al. (2012). In our study, by means of modified SAS processing, we only showed the Eu nanoparticles in second coating, forming a smooth layer over the chitosan’s rough coat (see Fig. 4).

Calcium alginate is anionic (i.e., negatively charged) and can attract positive charged chitosan. So the first coating layer of beads will be established by ionic bonds. In the next stage negative charged Eu S100 nanoparticles will attach the chitosan with the same mechanism (ionic bonds) and finally double coated beads are created (Badhana et al. 2013; Boeris et al. 2009; Kouchak et al. 2015). It should be also noted that beads made of calcium alginate (by extrusion method) are porous and the double layer coating with chitosan and Eu S100 nanoparticles can cover the porous beads and increase protection for the probiotic bacteria.

Bacterial counts decreased significantly during the study period (P < 0.001). In the case of *L. rhamnosus* the rate of this decline was significantly faster in free form in comparison with single (P = 0. 002) and double coated (P < 0.001) form and double coated bacteria were more resistant than single coated form (P = 0. 001). The differences between single and double coated was not statistically significant in *L. acidophilus* (P = 0.127), but the rate of decline in bacterial count in free form was significantly higher than single coated (P = 0.002) and double coated bacteria (P = 0.001). In conclusion, microencapsulation with alginate-chitosan-Eu S100 nanoparticles is a novel and efficient method for better viability of *L. acidophilus* and *L. rhamnosus* bacteria. Nevertheless, more studies are required to assess the viability of further probiotic strains as well as examine the guard effect of encapsulation and coating on the probiotic viability in animal and human models.

## Additional file

**Additional file 1: Table S1.** Raw data of cell viability (CFU ml^−1^) of three forms of *Lactobacillus acidophilus* and *Lactobacillus rhamnosus* (free, mono layer and double layer) after treatment in simulated gastrointestinal conditions.

## Abbreviations

GI: gastrointestinal
Eu: eudragit
IROST: Iranian Research Organization for Science and Technology
SAS: supercritical antisolvent technique
PDI: polydispersity index
CFU: colony forming units
SEM: scanning electron microscope
